# Inappropriate causal assumptions underlie Killingsworth, Kahneman, and Mellers’ conclusions

**DOI:** 10.1073/pnas.2313712121

**Published:** 2024-10-31

**Authors:** Julia M. Rohrer, Sebastian E. Wenz

**Affiliations:** ^a^Wilhelm Wundt Institute for Psychology, Leipzig University, Leipzig 04109, Germany; ^b^Knowledge Exchange and Outreach, GESIS–Leibniz Institute for the Social Sciences, Cologne 50667, Germany

Killingsworth, Kahneman, and Mellers’ [KKM, (1)] attempt to resolve their dispute about whether money can buy happiness is laudable. Their contribution shifts the focus from averages to other distribution features and measurement problems. Through piecewise linear quantile regressions, they discover that all assessed quantiles of happiness increase with log(income) until $100,000. Beyond $100K, lower quantiles do not increase significantly with log(income), suggesting a widening happiness distribution.

**Fig. 1.**
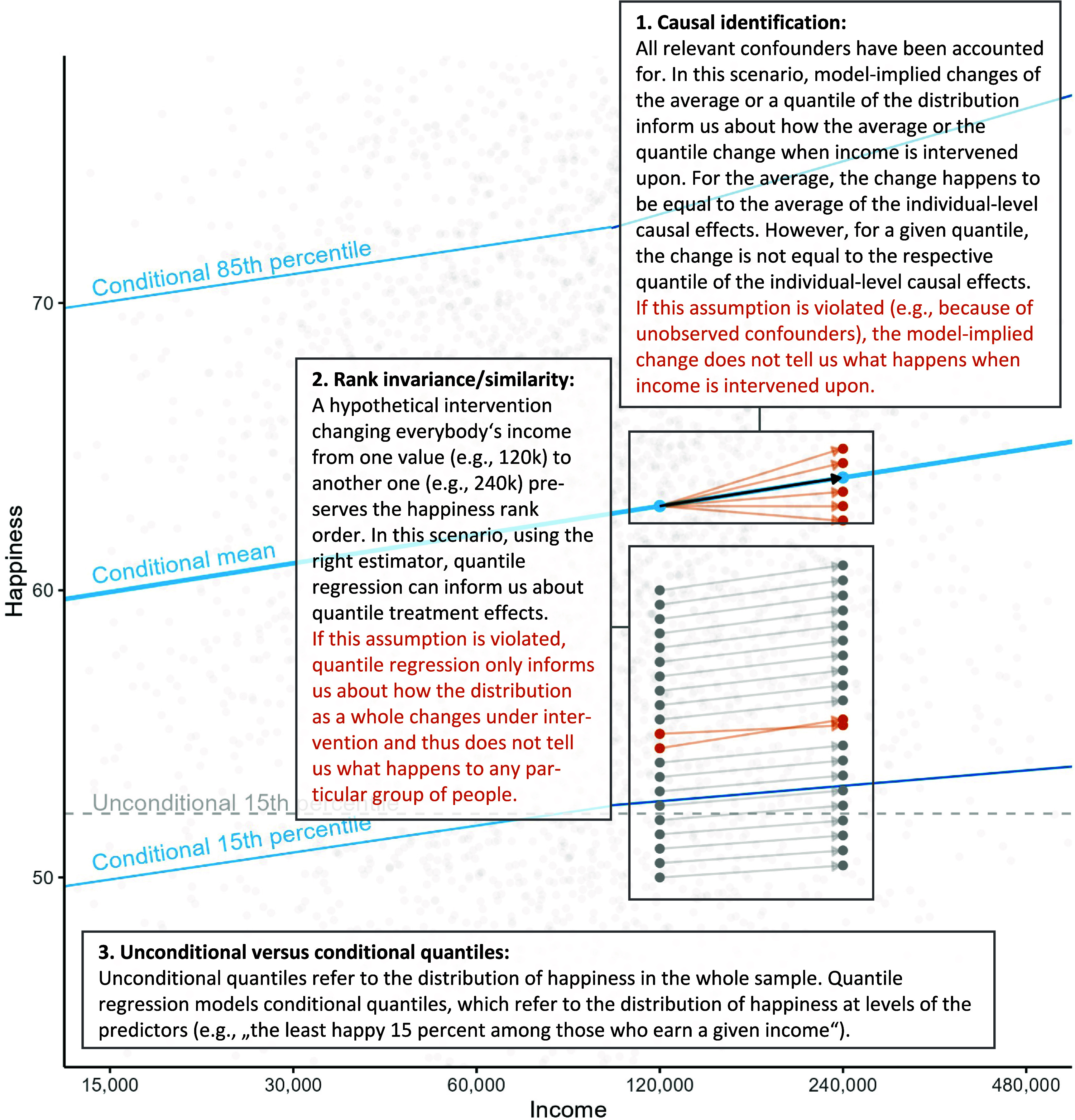
Visualization of two central assumptions (boxes 1 and 2) and a key distinction (box 3) for the correct interpretation of quantile regression results as demonstrating that “the suffering of the unhappy group diminishes as income increases up to 100K but very little beyond that.” Data were simulated to match the slopes reported by KKM. The *Upper* and *Lower* solid blue lines indicate the slopes of quantile regression models for the respective conditional quantiles; the solid blue line in the *Middle* indicates the slope of a linear regression (i.e., conditional mean model). The dashed gray line serves as a reference for the comparison of conditional and unconditional quantiles and simply shows where the 15th percentile of the unconditional outcome distribution is located in this simulated dataset.

“Do larger incomes make people happier?” reads like a question regarding the causal effect of income on happiness, and KKM provide the answer that “the suffering of the unhappy group diminishes as income increases up to 100K but very little beyond that.” For this to be true, the corresponding quantile treatment effects need to be *causally identified* (Fig. 1, box 1), and *rank invariance/similarity* (Fig. 1, box 2) needs to hold.

Considering causal identification, KKM state that they use “terms such as ‘increase’ and ‘gain’ for the ease of exposition” while only describing cross-sectional associations. However, cross-sectional associations only inform us about the diminishment of suffering in the absence of unobserved confounding. Given that KKM do not take into account *any* covariates, assuming the absence of confounding is not warranted.

Let us nonetheless assume successful causal identification. KKMs quantile regression models the conditional quantile function: A slope coefficient indicates the amount of change in a specific conditional quantile of the outcome (Fig. 1, box 3)—e.g., the 15th quantile, also called the 15th percentile, of happiness given income—associated with a one-unit change in the predictor (2). Quantile regression only supports statements about effects in “the unhappy group” under the assumption of *rank invariance*/*similarity*. This means that, as income is changed, individuals’ rank in the happiness distribution does not change systematically. However, people’s problems can be alleviated by money to varying degrees, and so rank invariance is not a plausible assumption.

What quantile regression does *not* do is simply estimate regressions in particular subgroups of people—such as the 15% least happy. In fact, without both causal identification and rank invariance, it does not refer to any specific group of people to begin with; it only describes the conditional outcome distribution (2).

KKM write that “practices that are standard in social science […] should be questioned more often.” We agree and suggest that such questioning should start with the widespread practice of presenting statistical results as if they could elucidate substantive disputes, without concern for the connection between statistics and substance and without attention to the necessary assumptions (3). Disclaimers such as “we are simply describing cross-sectional associations” are common (4), but they do little to prevent causal interpretations when the motivating research question is causal. Social scientists may be used to the delicate dance between causal and noncausal language and simply ignore the framing while absorbing the statistical facts. However, PNAS has a broad audience, and the media coverage of KKM attests to the fact that it is all too easy to jump from statistics to substance without justification.
